# The Comparative Effect of Different Timings of Whole Body Cryotherapy Treatment With Cold Water Immersion for Post-Exercise Recovery

**DOI:** 10.3389/fspor.2022.940516

**Published:** 2022-07-06

**Authors:** Adnan Haq, William J. Ribbans, Erich Hohenauer, Anthony W. Baross

**Affiliations:** ^1^Sports Studies, Moulton College, Moulton, United Kingdom; ^2^Sport and Exercise Science, University of Northampton, Northampton, United Kingdom; ^3^School of Health, Sport and Professional Practice, University of South Wales Sport Park, Pontypridd, United Kingdom; ^4^The County Clinic, Northampton, United Kingdom; ^5^Department of Business Economics, Health and Social Care, University of Applied Sciences and Arts of Southern Switzerland, Landquart, Switzerland

**Keywords:** whole body cryostimulation, muscle damage, sport, eccentric, cold, protocol

## Abstract

Despite several established benefits of Whole Body Cryotherapy (WBC) for post-exercise recovery, there is a scarcity of research which has identified the optimum WBC protocol for this purpose. This study investigated the influence of WBC treatment timing on physiological and functional responses following a downhill running bout. An additional purpose was to compare such responses with those following cold water immersion (CWI), since there is no clear consensus as to which cold modality is more effective for supporting athletic recovery. Thirty-three male participants (mean ± SD age 37.0 ± 13.3 years, height 1.76 ± 0.07 m, body mass 79.5 ± 13.7 kg) completed a 30 min downhill run (15% gradient) at 60% VO_2_ max and were then allocated into one of four recovery groups: WBC1 (*n* = 9) and WBC4 (*n* = 8) underwent cryotherapy (3 min, −120°C) 1 and 4 h post-run, respectively; CWI (*n* = 8) participants were immersed in cold water (10 min, 15°C) up to the waist 1 h post-run and control (CON, *n* = 8) participants passively recovered in a controlled environment (20°C). Maximal isometric leg muscle torque was assessed pre and 24 h post-run. Blood creatine kinase (CK), muscle soreness, femoral artery blood flow, plasma IL-6 and sleep were also assessed pre and post-treatment. There were significant decreases in muscle torque for WBC4 (10.9%, *p* = 0.04) and CON (11.3% *p* = 0.00) and no significant decreases for WBC1 (5.6%, *p* = 0.06) and CWI (5.1%, *p* = 0.15). There were no significant differences between groups in muscle soreness, CK, IL-6 or sleep. Femoral artery blood flow significantly decreased in CWI (*p* = 0.02), but did not differ in other groups. WBC treatments within an hour may be preferable for muscle strength recovery compared to delayed treatments; however WBC appears to be no more effective than CWI. Neither cold intervention had an impact on inflammation or sleep.

## Introduction

The extreme cold of Whole Body Cryotherapy (WBC) has become an emerging tool for sport and exercise recovery (Lombardi et al., 2017), demonstrating reductions in pain (Hausswirth et al., 2011), inflammation (Pournot et al., 2011), improved muscle strength recovery (Haq et al., 2021) and benefits to range of motion (De Nardi et al., 2015). Despite such effects, there is currently limited research which has identified conclusively the optimum WBC protocol for such purposes. Further, due to the differences associated with various sports, as well as diversity in training backgrounds, it might be pertinent to individualize cryotherapy treatments. WBC is also an expensive and relatively unique treatment that is not easily accessible for the majority of the athletic population. Therefore, it is necessary to determine optimal cryotherapy treatments for maximal impact and financial prudence.

Whilst WBC treatments are typically 3 min duration at −110°C to −140°C (Lombardi et al., 2017), the influence of treatment timing on WBC effectiveness has not been addressed. It would be useful to determine at what stage of recovery post-exercise WBC still evokes beneficial effects. Due to the specific mechanisms of exercise-induced muscle damage (EIMD) and breakdown in the first few hours post-exercise, recovery interventions applied after several hours may be too late to effectively intervene. For instance, the first 4 h of post-exercise recovery is characterized by neutrophil infiltration, release of reactive oxygen species and muscle cell lysis and necrosis, whereas during subsequent hours, a pro-inflammatory macrophage and cytokine response typically dominates (Peake et al., 2017). Studies reporting the positive effect of WBC on muscle recovery have applied treatments from within 15 min post-exercise (Hausswirth et al., 2011; Ferreira-Junior et al., 2014, 2015), up to 45 min (Fonda and Sarabon, 2013) and 1 h post-exercise (Kruger et al., 2015). Applying the treatment 24 h post-exercise has not resulted in beneficial effects on muscle torque or soreness (Costello et al., 2012a). It is conceivable that the 24-h period after exercise represents a window when cryotherapy can intervene to influence muscle damage progression, thus a longer delay in the treatment may be ineffective in mitigating muscle damage to benefit recovery. Factors such as cool down, transport and treatment accessibility may present additional logistical challenges for athletes wishing to use cryotherapy. Research on the impact of delayed treatments beyond 1 h is lacking and no study has yet to assess the influence of WBC treatment timing on the damage or recovery response to exercise.

Another contentious area within the field of cryotherapy is the comparative effect of WBC against cold water immersions (CWI), especially considering the high popularity and widespread use of the latter (Allan et al., 2021), as well as its accessibility and affordability. Whilst WBC has the advantage of imposing more extreme temperature on its users, thereby creating a larger temperature gradient between the body surface and the surroundings, CWI creates a hydrostatic effect, which has been argued to augment the effect of alleviating muscle swelling (White and Wells, 2013). Furthermore, cold water has a higher thermal conductivity than cold air, which has a higher potential to extract more heat from the body (Bleakley et al., 2014). Several effects of CWI have already been established post-exercise, particularly with regards to mitigating muscle damage markers such as muscle strength reductions and soreness (Bailey et al., 2007; Ascensão et al., 2011; Rossato et al., 2015). Previous studies that have directly compared the effects of WBC with CWI on recovery parameters post-exercise reveal contrasting findings (Abaïdia et al., 2017; Hohenauer et al., 2017, 2020; Wilson et al., 2018, 2019; Qu et al., 2020). Further research is warranted in this area to compare these two cryotherapy methods on the effects of damage and recovery markers post-exercise.

This study aims to build on findings from previous work (Haq et al., 2021) by exploring the comparative effects of two different timings of WBC on EIMD following a downhill run, as well as comparing WBC with CWI. Downhill running is a commonly utilized eccentric exercise protocol for inducing muscle damage that remains under-investigated in the field of cryotherapy. As well as muscle damage markers, additional parameters to be assessed in this study include limb blood flow, inflammation and sleep which could collectively provide further insight on the mechanisms of post-exercise recovery following cryotherapy. For instance, the importance of sleep for athletic recovery has already been established (Walsh et al., 2020) and studies have indicated beneficial effects of WBC on sleep quality (Douzi et al., 2018, 2019). Clarifying a preferred timing of treatment may also assist athletes and coaches in determining an optimum protocol for WBC, which could represent a significant step in the constant strive for performance advantages.

Consequently, the objectives of this study were two-fold: firstly, to examine the influence of different timings of WBC treatment on recovery parameters post-downhill run; secondly, to compare the effects of WBC with CWI on recovery parameters post-downhill run.

## Materials and Methods

### Participants

The sample size calculation (G^*^Power, Franz Faul University Kiel, Germany) was performed using the expected effect sizes from a comparable study (Hohenauer et al., 2020). The following design specifications were taken into account: significance level 0.05, power 0.8, effect size 0.4. The sample size estimated according to these specifications revealed that 28 participants would be appropriate. Thirty-three male volunteers (mean ± SD age 37.0 ± 13.3 years, height 1.76 ± 0.07 m, body mass 79.5 ± 13.7 kg) completed this study. Participants were randomly assigned into four groups: WBC1 (*n* = 9), WBC4 (*n* = 8), CWI (*n* = 8), and control (CON, *n* = 8). All participants were of a suitable fitness level, partaking in regular physical activity. Sample characteristics for each group are summarized in Table 1. Participants provided written informed consent prior to assessment. Ethical approval was obtained from the University of Northampton Graduate School Research Ethics Committee.

**Table 1:** Summary of characteristics for whole body cryotherapy 1 h (WBC1), 4 h (WBC4), cold water immersion (CWI), and control (CON) participants.

	**WBC1** **(*n* = 9)**	**WBC4** **(*n* = 8)**	**CWI** **(*n* = 8)**	**CON** **(*n* = 8)**	**OVERALL** **(*n* = 33)**
Age (yrs)	35.3 ± 14.9	27.8 ± 5.9	38.1 ± 12.5	47.2 ± 12.0	37.0 ± 13.3
Height (m)	1.76 ± 0.08	1.76 ± 0.08	1.78 ± 0.05	1.73 ± 0.04	1.76 ± 0.07
Body mass (kg)	89.5 ± 20.8	75.8 ± 8.3	77.6 ± 7.0	74.0 ± 8.2	79.5 ± 13.7
Body mass index (kg/m^2^)	28.6 ± 4.9	24.6 ± 2.5	24.6 ± 2.1	24.7 ± 2.6	25.7 ± 3.6
Body fat %	23.2 ± 7.0	18.0 ± 3.2	21.1 ± 4.3	21.7 ± 3.9	21.1 ± 5.1
Absolute VO_2_ max (l/min)	3.52 ± 0.4	3.66 ± 0.42	3.40 ± 0.6	3.35 ± 0.24	3.41 ± 0.44
Relative VO_2_ max (ml/min/kg)	40.4 ± 6.1	43.2 ± 7.7	44.2 ± 5.1	45.3 ± 3.86	43.3 ± 5.4

*Data presented as mean ± SD*.

### Experimental Design

This study adopted a randomized independent groups design, using a similar protocol as a previous study in this line of work (Haq et al., 2021). There were three laboratory trials (ambient temperature 20 ± 0.5°C) and all participants were asked to refrain from alcohol and strenuous exercise for 24 and 48 h, respectively, prior to each trial.

For the first trial, participants' anthropometric characteristics were assessed. Skinfold calipers (Harpenden Indicators, UK) were used to estimate body fat percentage using the four site method described previously (Haq et al., 2021). Participants were familiarized to a muscle torque assessment *via* three contractions using a Biodex dynamometer: two submaximal isometric contractions (60 and 80% effort), followed by a singular maximal contraction. Maximal aerobic capacity (VO_2_ max) was assessed using an incremental treadmill protocol, as described previously (Haq et al., 2021). Absolute and relative VO_2_ max values were reported and 60% of the absolute VO_2_ max was calculated. Participants completed a separate maximal muscle torque assessment. Participants were provided a sleep watch (Fitbit Inspire, California) and a sleep questionnaire, before leaving the laboratory.

**Figure 1:**
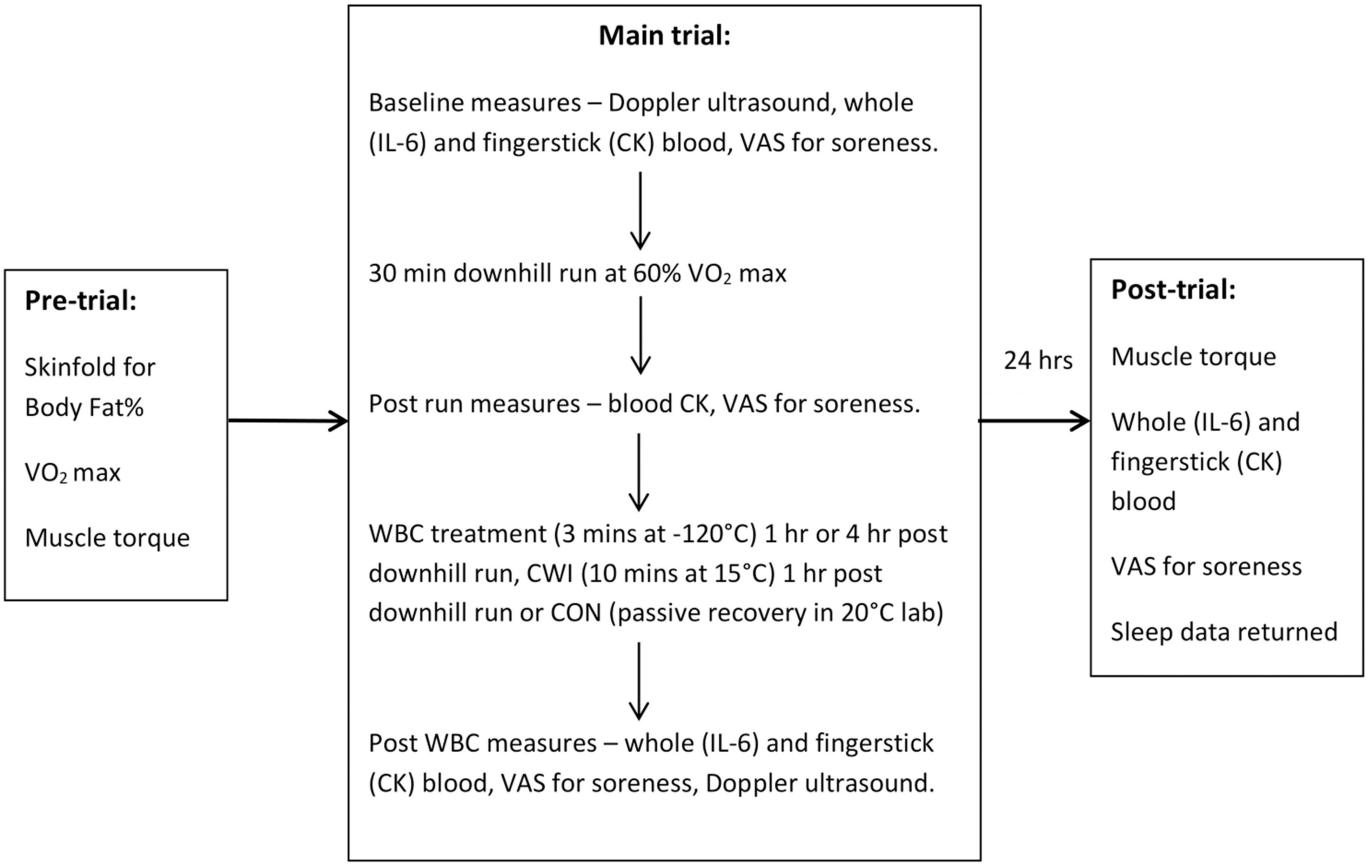
Protocol summary of measures for each trial. Sleep was also assessed for 3 consecutive nights prior to main trial and night following. WBC, Whole Body Cryotherapy; CWI, Cold Water Immersion; CON, Control; CK, creatine kinase; VAS, visual analog scale.

Within 2 weeks of the first trial, participants performed their main trial. Participants arrived in a physically rested and hydrated state and were instructed to avoid caffeine for 4 h prior to the session. Participants followed the protocol involving the downhill run, cryotherapy intervention and measurements of their variables pre and post-run, as specified in Figure 1.

Participants returned to the laboratory 24 h after the downhill run for their final assessments, which included their post muscle torque assessment following the same protocol as the pre-trial. The sleep watch and questionnaire was also returned for subsequent analysis of sleep data.

The study protocol is summarized in Figure 1.

### Muscle Damaging Protocol

Participants performed a 30 min downhill run on a treadmill (HPCosmos, Germany) at a 15% decline using the procedures described previously (Haq et al., 2021). Target heart rate was predetermined from the VO_2_ max vs. heart rate relationship so that a running intensity corresponding to 60% of their VO_2_ max was maintained.

### Recovery Interventions

Whole Body Cryotherapy treatments occurred during the main trial and were undertaken in a two-stage cryogenic chamber (JUKA, Poland). Participants were screened for contraindications following the completion of a health questionnaire, including hypertension, other cardiovascular diseases, open wounds, cold intolerance and neural/mental disorders. Participants wore the appropriate protective clothing and followed the same procedure as described previously (Haq et al., 2022). Participants entered the cryotherapy chamber, initially exposed to a vestibule chamber at −60°C for 30 s, followed by the main chamber at −120°C for 150 s. On completion, the exit door for the main chamber opened and the participant exited. Thereafter, participants were advised to stay mobile before changing in usual clothing.

WBC1 participants were transported to a therapy center for their WBC treatment 60 min post-downhill run. WBC4 participants were allowed to leave the laboratory and instructed to arrive at the therapy center ahead of their scheduled WBC treatment 4 h post-downhill run, whilst maintaining hydration status and avoiding caffeine, alcohol, exercise and other recovery modalities.

Cold water immersion participants underwent treatment 1 h post-downhill run in the same building as the laboratory. Participants were immersed for 10 min into a plastic 200 L butt filled with water up to the iliac crest. The water temperature was maintained at 15 ± 0.5°C using a thermometer and the addition/removal of ice. This temperature lies within the 10–15°C range that the majority of CWI studies have used revealing benefits post-exercise (Versey et al., 2013). Due to height differences, some participants were instructed to slightly adjust their position whilst standing in the water to ensure immersion up to their iliac crest. Participants were also instructed to avoid excessive movements throughout the immersion.

Control participants did not undergo cold treatment post-downhill run and had the same variables assessed under controlled laboratory conditions at the same corresponding time points as the WBC and CWI groups.

### Maximal Muscle Torque

Isometric maximal torque of the right quadriceps was assessed by an isokinetic dynamometer (Biodex Medical Systems 3, New York, USA) calibrated prior to the study. The dynamometer was fitted with a lever arm attachment locked in at 90° leg extension. Participants sat on the chair using the setup described previously (Haq et al., 2021). Participants performed two warm up contractions at 60 and 80% effort, respectively, by exerting force against the pad. Following 2 min rest, they performed four maximal contractions (with 2 min recoveries) with verbal encouragement given throughout (Baross et al., 2013). All contractions were 5 s in duration. The peak torque (Nm) was determined as the maximum of the four contractions. A pilot study conducted in the laboratory revealed a day to day variance of 5.3% within individuals.

### Muscle Soreness

Participants' muscle soreness was assessed *via* a visual analog scale (VAS) using the wall squat procedure (Haq et al., 2021). Participants' knees were flexed to a 90° angle, holding the position for 3 s. Participants marked on the scale how much pain they felt in their upper legs from “no pain” to “pain as bad as it could possibly be”. The distances marked in millimeters were converted into percentages.

### Creatine Kinase

Participants provided a 30 μl fingerstick blood sample for the measurement of CK levels, using a test strip inserted into a Reflotron Plus analyser (Oberoi Consulting, Derby), as described previously (Haq et al., 2021).

### Femoral Artery Blood Flow

Participants were rested in a supine position for at least 5 min before images were taken. All measurements were taken by the same operator to minimize inter-rater experimental bias. Resting femoral artery blood flow was assessed *via* ultrasound using a 12L-RS linear transducer probe (frequency range 5–13 MHz) attached to an ultrasound machine (Vivid I, GE Medical Systems, Israel) in the pulse wave Doppler mode. Images were taken at an insonation angle of 60° at the superficial femoral artery of the right thigh ~3 cm inferior from the bifurcation with the deep femoral artery. Longitudinal B-mode images of the lumen-arterial wall interface were optimized. Each measurement in the pulse wave was recorded for 30 s. Images were stored and later analyzed. Arterial diameter was measured perpendicularly between the lumen-intima interfaces of the superior and inferior walls of the artery (Baross et al., 2012). Arterial diameters and velocities were determined as the mean of three 30 s pulse wave measures. Mean blood velocities of each trace were measured from the velocity time integrals on the machine.

Overall leg blood flow (LBF) was calculated as follows:

LBF (ml/min) = 60 × Mean blood velocity (cm/s) × π × artery radius^2^ (Blanco, 2015).

### Interleukin-6

Whole blood samples were obtained by venepuncture into 6 ml EDTA vacutainer tubes (BD Diagnostics, Oxford). Following separation *via* centrifuge at 2,000 g for 10 min, plasma was transferred into 1.5 ml eppendorf tubes and frozen at −80°C for subsequent analysis. Concentrations of IL-6 in thawed samples were quantified by Bio-Plex Multiplex Immunoassays (Bio-Rad, UK), a technique that utilizes dual fluorescent polystyrene beads. The IL-6 detection limit was 0.49 pg/ml. A high reliability of the multiplex immunoassay system has previously been demonstrated, with mean co-efficient of variance (CV) values as low as 2.8% (Tighe et al., 2013).

### Sleep

Sleep was assessed using motion and sleep tracking wrist watches (Fitbit Inspire, California) for four consecutive nights—three nights prior to the downhill run trial and the night following. The main variable of interest was sleep efficiency:

Sleep efficiency (%) = (Total time in bed – time awake)/Total time in bed

Total time in bed was determined as the “sleep duration” displayed on the watch data. The watch also provided the number of awakenings and time spent awake. Participants were instructed to wear the watches from the time they went to bed and remove them upon waking. Compared to polysomnography reference values, Fitbit watches have been reported to overestimate sleep efficiency by 2–15% and underestimate time spent awake by 6–44 min, with no significant difference in sleep onset latency (*p* = 0.37) (Haghayegh et al., 2019). Compared to Sensewear or Actiwatch accelerometers, measurement errors of within ±10% were reported for time in bed and time spent asleep (Feehan et al., 2018).

A sleep questionnaire was also completed for the same nights where participants assessed their overall sleep quality on a scale from 5-very good to 1-very poor.

### Statistical Analysis

All data was analyzed using SPSS Version 26. Data for all variables was assessed for normal distribution by the Shapiro-Wilk test. Data was transformed as appropriate (log or square root) when data significantly deviated from normal distribution. Muscle torque, femoral blood flow, IL-6 and sleep efficiency were log transformed. Muscle soreness and CK were square root transformed. Two way repeated measures ANOVAs were used to assess the interaction effect between treatment group (WBC1, WBC4, CWI, and CON) and time for all major variables (muscle torque, femoral artery blood flow, IL-6, sleep, soreness, CK). Muscle torque and femoral artery blood flow was assessed with a 4 (group) × 2 (time) interaction; IL-6 was assessed with a 4 × 3; soreness, CK and sleep were assessed with a 4 × 4. Paired *t*-tests and *post-hoc* analyses using Bonferroni corrections were applied within groups where appropriate to examine differences between specific timepoints. Effect sizes (Cohen's d) were calculated for muscle torque, the main dependent variable of interest, and defined as follows: small – 0.2; medium – 0.5; large – 0.8 (Cohen, 1992). Significance levels were set at 0.05.

## Results

Original data (pre-transformation) are presented in figures.

### Muscle Torque

**Figure 2:**
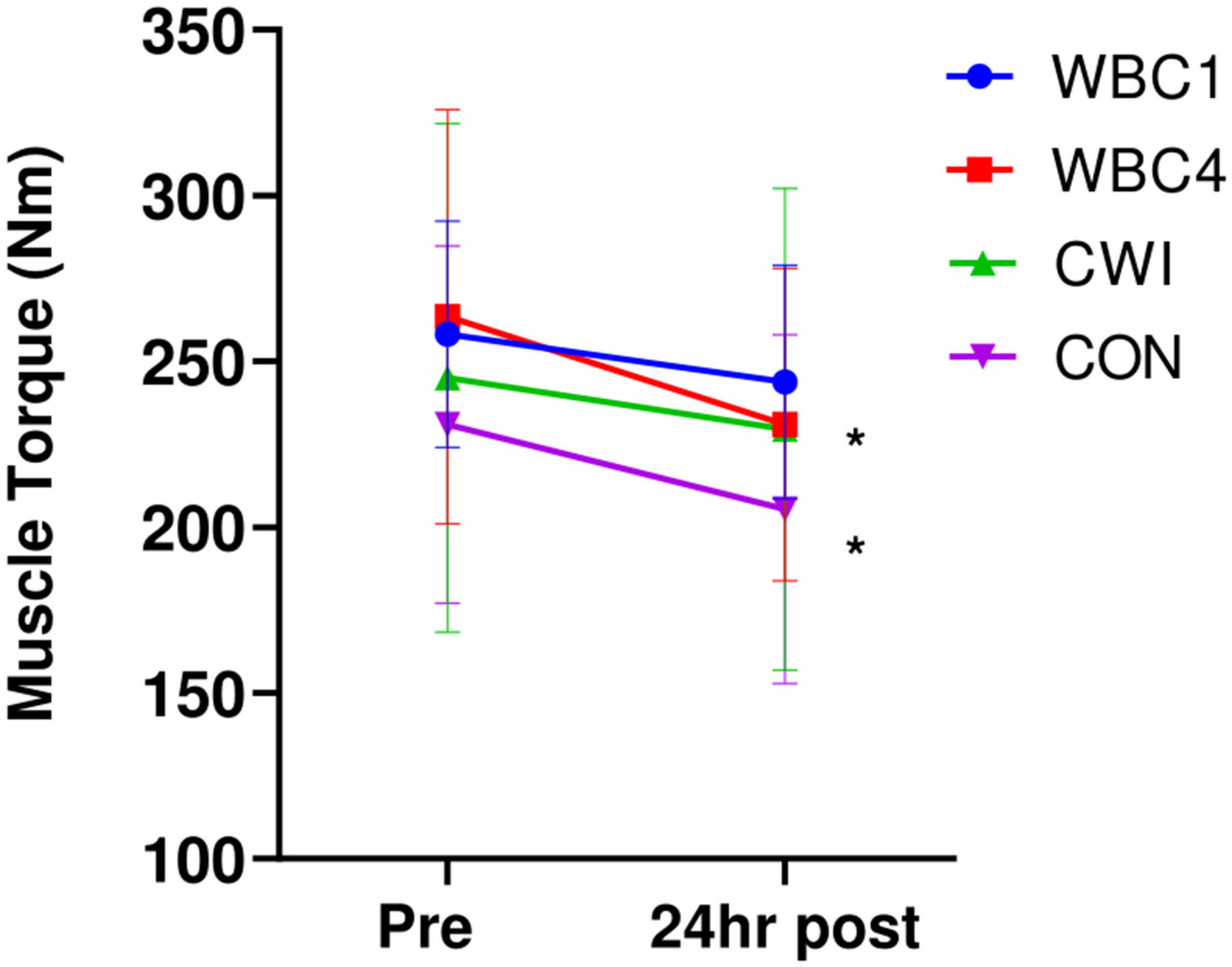
Maximal muscle torques for WBC1, WBC4, CWI and CON groups. *p < 0.05 for decrease in WBC4 and CON only. Data presented as means ± standard deviations. *N* = 33.

There were no between-group differences in pre-muscle torque (all pairwise comparisons *p* > 0.6). There was a significant decrease in maximal isometric torque 24 h post-downhill run for the WBC4 and CON groups (WBC4, 263.5 ± 62.5 Nm vs. 231.0 ± 47.2 Nm, *p* = 0.04, *d* = 0.59; CON, 230.9 ± 53.9 Nm vs. 205.4 ± 52.6 Nm, *p* = 0.00, *d* = 0.48), a slight decrease for the WBC1 group that approached significance (258.2 ± 34.2 Nm vs. 243.8 ± 35.3 Nm, *p* = 0.06, *d* = 0.42) and no significant decrease for CWI (245.1 ± 76.8 Nm vs. 229.5 ± 72.7 Nm, *p* = 0.15, *d* = 0.21). The mean torque decreases were 14.4 ± 20.2 Nm (5.6%), 32.5 ± 37.0 Nm (10.9%), 15.6 ± 27.1 Nm (5.1%) and 25.5 ± 14.5 Nm (11.3%) for the WBC1, WBC4, CWI, and CON groups, respectively (Figure 2), with no differences between groups (*p* = 0.45) or group^*^time interaction (*p* = 0.45).

### Muscle Soreness

**Figure 3:**
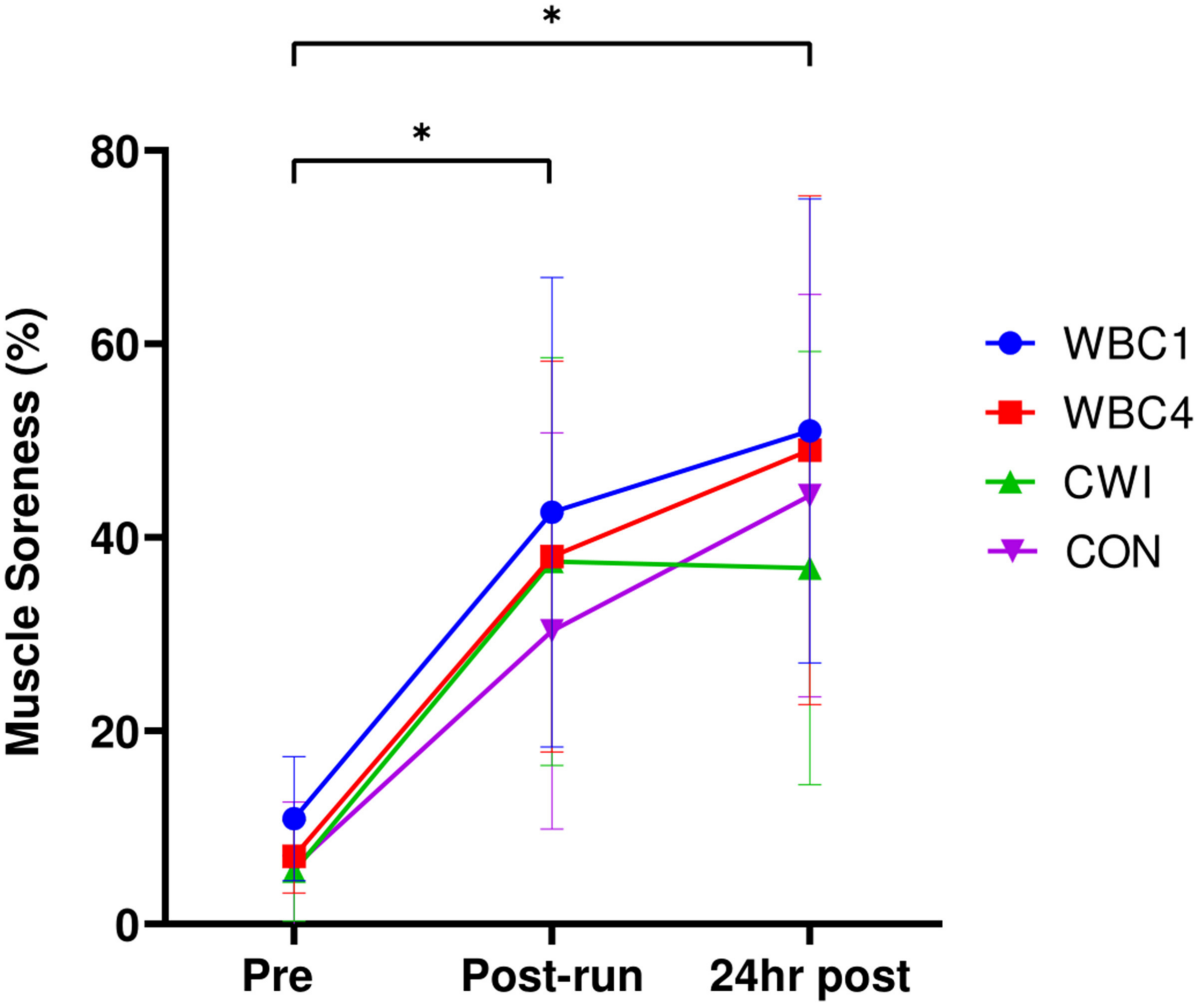
Maximal muscle torques for WBC1, WBC4, CWI and CON groups. *p < 0.05 for decrease in WBC4 and CON only. Data presented as means ± standard deviations. *N* = 33.

Soreness significantly increased from baseline to post-downhill run and 24 h post-run for all groups (overall effect of time *p* = 0.00 for all groups, Figure 3), with a peak reached at 24 h for WBC1 (51%), WBC4 (49%), and CON (44%). The peak soreness for CWI was obtained post-downhill run (38%). There was no group^*^time interaction effect (*p* = 0.87). Pairwise comparisons revealed no differences between any of the groups at 24 h post-run (all *p*-values > 0.6).

### Creatine Kinase

**Figure 4:**
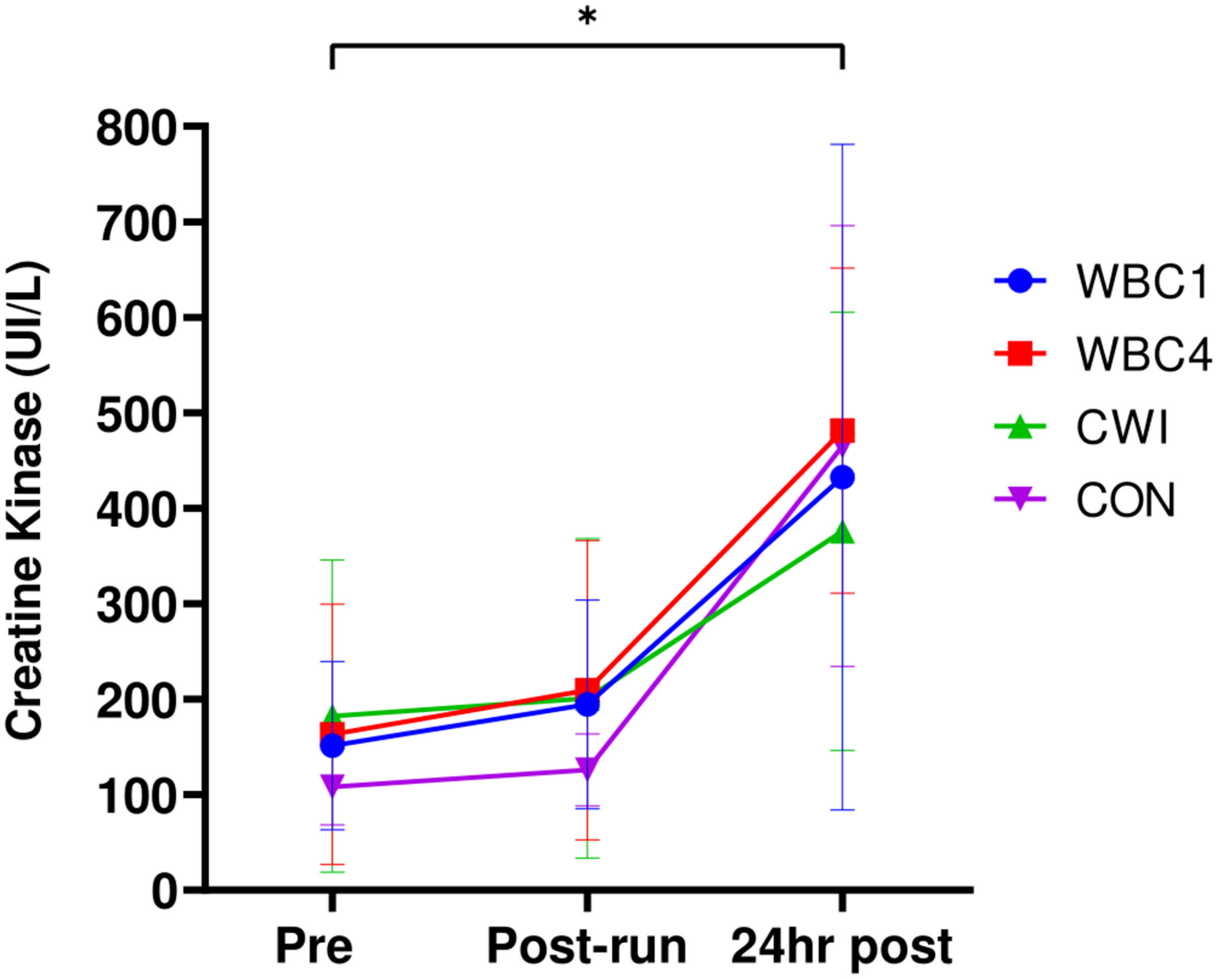
Blood CK values for WBC1, WBC4, CWI and CON groups. *p < 0.05 for increase at 24 h post for all groups. Data presented as means ± standard deviations. *N* = 33.

Blood CK significantly increased from baseline at 24 h post-run in all four groups (WBC1: 151.2 ± 88.2 IU/L vs. 442.8 ± 327.4 IU/L; WBC4: 163.1 ± 136.6 IU/L vs. 481.4 ± 170.5 IU/L; CWI: 182.5 ± 163.8 IU/L vs. 375.8 ± 229.8 IU/L; CON: 108.2 ± 39.9 IU/L vs. 465.3 ± 230.9 IU/L, *p* = 0.00 for all groups, Figure 4). However, there were no group^*^time interaction effects (*p* = 0.25) with no difference noted between groups at post 24 h (*p* = 0.76).

### Femoral Artery Blood Flow

Femoral artery blood flows are displayed in Table 2. There was no difference in blood flow for WBC1, WBC4, or CON groups (all *p*-values > 0.5). Blood flow significantly decreased for the CWI group (*p* = 0.02) whilst there was no significant group^*^time interaction effect (*p* = 0.19).

**Table 2:** Femoral artery blood flows for WBC1, WBC4, CWI, and CON groups.

	**Pre (ml/min)**	**Post-run/cryo (ml/min)**
WBC1	244.5 ± 50.5	248.8 ± 53.7
WBC4	235.4 ± 102.0	258.5 ± 161.0
CWI	195.0 ± 59.6	158.6 ± 80.3^*^
CON	206.2 ± 53.9	214.3 ± 59.7

*Data presented as means ± standard deviations. N = 33*.

* *p < 0.05 for decrease in CWI group*.

### Plasma IL-6

IL-6 results are displayed in Table 3. The majority of samples were below the detectable limit of 0.49 pg/ml. These samples were recorded as 0.25 pg/ml for analysis purposes. There were no significant increases in IL-6 post-run for any of the groups (*p* > 0.10 for all groups) and there was no group^*^time interaction (*p* = 0.43).

**Table 3:** Plasma IL-6 response for WBC1, WBC4, CWI, and CON groups.

	**Pre**	1 h post-run/post-cryo^*^	**24 h post**
WBC1	0.30 ± 0.13 pg/ml	0.47 ± 0.41 pg/ml	0.25 ± 0.00 pg/ml
WBC4	0.32 ± 0.18 pg/ml	0.59 ± 0.31 pg/ml	0.25 ± 0.00 pg/ml
CWI	0.25 ± 0.00 pg/ml	0.32 ± 0.15 pg/ml	0.25 ± 0.00 pg/ml
CON	0.25 ± 0.00 pg/ml	1.02 ± 0.88 pg/ml	0.25 ± 0.00 pg/ml

*Data presented as means ± standard deviations. N=33*.

* *Measurement for WBC4 group taken post-WBC (~4 h post-run)*.

### Sleep

Average durations in bed and sleep efficiency percentages each night are displayed in Table 4, along with sleep questionnaire ratings. There was no difference within or between groups for sleep efficiency (effect of time *p* > 0.4 for all groups; group^*^time interaction effect, *p* = 0.39). There was no difference within or between groups for sleep questionnaire ratings (effect of time *p* > 0.2 for all groups; group^*^time interaction effect, *p* = 0.62).

**Table 4:** Sleep durations, % efficiency and questionnaire ratings over 4 consecutive nights for WBC1, WBC4, CWI, and CON groups.

**Variable**		**Night 1**	**Night 2**	**Night 3 (night before main trial)**	**Night 4 (night after main trial)**
Sleep duration (min)	WBC1	469.0 ± 74.0	406.1 ± 79.2	393.3 ± 105.7	461.1 ± 52.6
	WBC4	380.0 ± 86.5	421.4 ± 80.8	362.4 ± 68.3	405.8 ± 40.5
	CWI	484.8 ± 137.0	481.2 ± 69.2	495.4 ± 83.3	530.2 ± 129.2
	CON	417.0 ± 90.1	456.3 ± 44.6	452.5 ± 35.0	466.8 ± 54.6
Sleep efficiency	WBC1	95.0% ± 2.6	93.7% ± 2.6	93.7% ± 3.1	94.5% ± 2.9
	WBC4	94.2% ± 3.3	95.7% ± 3.5	96.5% ± 1.8	92.4% ± 8.1
	CWI	90.0% ± 14.5	87.3% ± 19.4	87.6% ± 16.8	91.5% ± 10.4
	CON	97.0% ± 2.6	97.2% ± 2.2	97.0% ± 2.4	97.0% ± 2.0
Sleep questionnaire	WBC1	3.1 ± 0.9	3.2 ± 0.8	3.1 ± 0.9	3.4 ± 1.1
	WBC4	2.7 ± 1.0	3.3 ± 1.5	3.2 ± 0.8	4.0 ± 0.6
	CWI	3.1 ± 0.7	3.3 ± 0.5	3.4 ± 0.8	3.7 ± 0.5
	CON	3.4 ± 1.0	3.7 ± 0.5	3.0 ± 0.6	± 0.5

*Sleep questionnaire scores: 5- “very good,” 1- “very poor”. Downhill run and cryotherapy occurred between nights 3 and 4. Data presented as means ± standard deviations*.

## Discussion

The principal aim of this study was to investigate the potential influence of WBC treatment timing on responses to muscle damaging exercise, since no prior study had manipulated WBC timing as a protocol factor. The main finding was that timing did not have a significant impact on the outcomes following a downhill run, however there is evidence to suggest that taking cryotherapy treatments within an hour post-exercise may be preferable for muscle strength recovery than delaying by several hours. Secondarily, WBC did not appear to be any more beneficial than CWI for all the assessed variables. There was also no impact of either cold treatment intervention on sleep.

There was evidence that significant muscle damage occurred in the WBC4 and CON participants as observed by the significant decrease in muscle torque 24 h post-run (~11% decrease in these groups). The decrease of 5.6% at 24 h for the WBC1 group is comparable to the WBC cohort in a previous downhill run study (Haq et al., 2021). Meanwhile, there was no significant decrease for the CWI group (5.1%, *p* = 0.15), which would indicate that CWI can be a useful remedy to support post-exercise recovery, as supported by some earlier findings (Bailey et al., 2007; Rowsell et al., 2011). Since the slight decreases for WBC1 and CWI are very similar to the within subject day to day variance of 5.3%, this would suggest that the WBC1 and CWI groups responded better with regards to muscle strength recovery. This may support the theory that delaying WBC treatment by several hours is disadvantageous due to the time course of the muscle breakdown mechanisms post-exercise, such as infiltration of neutrophils, release of reactive oxygen species, as well as disruption of myofibrils, calcium handling and muscle contractile components (Peake et al., 2017). However, the lack of difference between the WBC1 and CWI groups indicates that WBC is no more effective than CWI despite the more extreme cold temperatures. This supports earlier findings revealing no significant recovery benefits of WBC treatments compared to CWI post-exercise (Hohenauer et al., 2017, 2020; Wilson et al., 2018).

Regarding muscle soreness, there did not appear to be an impact of WBC treatment timing since both WBC groups had near identical levels of soreness 24 h post-exercise. As reported previously (Haq et al., 2021), the potential limitation of not assessing soreness beyond 24 h should be considered. The lack of impact of WBC on muscle soreness post-downhill run corroborates the findings from previous work (Haq et al., 2021), since soreness post-WBC was no lower than CON. Although not significantly different from the other groups, CWI participants had the lowest peak of muscle soreness (38%, Figure 3) and were the only group where muscle soreness did not peak at 24 h post. This opposes findings from the two previous downhill run and CWI studies to date (Crystal et al., 2013; Rossato et al., 2015) where the peak soreness occurred at 24 h post-run. It is conceivable that the soreness for the CWI group would have dropped further at 48 h post-run, potentially favoring the use of CWI over WBC. A useful consideration is the clinically relevant difference in soreness. Clinically important differences in pain VAS scores could be as low as 13% (Gallagher et al., 2001; Camacho-Villa et al., 2021), which corresponds to the 13% difference in peak soreness between the WBC1 and CWI groups. Such differences could thereby have implications for subsequent exercise performance.

Whilst the impact of WBC on muscle soreness remains contentious (Costello et al., 2015), several findings have supported the use of CWI for alleviating soreness post-exercise (Bailey et al., 2007; Ascensão et al., 2011; L'Hermette et al., 2020). This is possibly due to the fact that cold water imposes a hydrostatic effect which can further curtail swelling and efflux of metabolites (White and Wells, 2013), as well as having a higher potential to extract heat from the body (Bleakley et al., 2014).

There was no impact of either cryotherapy intervention on plasma CK levels. The overall CK values at 24 h post were quite moderate albeit significantly elevated from baseline (all groups <500 I/L, Figure 4) and indicates the relatively mild extent of muscle damage caused by the downhill run. Whilst downhill running causes substantial muscle damage, the extent of damage is not as severe as other commonly adopted EIMD protocols such as drop jumps (Westerlund et al., 2009; Hohenauer et al., 2020) and isolated arm curls (Yoon and Kim, 2020). Neither cryotherapy intervention was successful in blunting the plasma CK response, regardless of treatment timing. It therefore appears that singular cryotherapy treatments (cold air or water) are insufficient to significantly alter blood CK levels post-exercise (using these specific protocols) and affect the muscle membrane breakdown following exercise.

There were no between group differences in femoral artery blood flow. However, whilst there was a significant decrease in leg blood flow for the CWI group post-treatment, there were no decreases for either WBC intervention. One proposed mechanism of WBC potentially aiding the alleviation of muscle damage is a reduced blood supply causing reduced muscle metabolism, thus reduced muscle breakdown (White and Wells, 2013; Ferreira-Junior et al., 2014). Although the impact of WBC on muscle metabolism *per se* remains inconclusive, previous studies have demonstrated reduced muscle temperatures and oxygenation post-WBC (Costello et al., 2012b; Hohenauer et al., 2020). In the current study there was no impact of WBC on femoral artery blood flow, regardless of the timing. One study has previously reported significant reductions in leg blood flow post-CWI and post-WBC but with more pronounced reductions following CWI (Mawhinney et al., 2017). One notable difference is that this prior study assessed femoral blood flow at more time points pre- and post-treatment. Due to the need to transport WBC participants in the current study, it was not feasible to measure leg blood flow between the downhill run and the cryotherapy treatment without further treatment delay. Nonetheless, the finding that leg blood flow significantly reduced following CWI post-run and not following WBC (possibly due to the hydrostatic pressure) might explain why CWI is potentially favorable for alleviating muscle soreness and/or swelling, perhaps due to lower metabolic demands (Ihsan et al., 2016). Muscle metabolism or swelling was not assessed in the current study, but the possibility of addressing these markers in further comparative studies between WBC and CWI remains a possible avenue for further research.

Despite the presence of muscle damage, the IL-6 results would indicate that the downhill run did not cause a substantial inflammatory response, with an average peak of 1.02 pg/ml for CON. This is in contrast with previous downhill run studies (Smith et al., 2007; Dolci et al., 2015) observing IL-6 values ranging from 3 to 12 pg/ml post-run. One potential cause of a lack of substantial inflammatory response could have been the mild extent of muscle damage (11% decrease in torque for CON, plasma CK <500 I/L). Additionally, downhill runs of longer durations than 30 min may induce more pronounced increases in inflammatory markers, as was evident in the aforementioned downhill run studies. Further research to clarify the potential impact of single cryotherapy treatments on inflammatory responses post-muscle damaging exercise would be informative.

Due to the established importance of sleep quality for athletic recovery and performance (Walsh et al., 2020), it would be pertinent to further explore the potential impact of post-exercise recovery interventions on sleep. There was no impact of either cryotherapy intervention on sleep quality. This contrasts previous WBC (Douzi et al., 2018) and CWI (Tabben et al., 2018) research. Purported claims for the potential benefits of cold applications on recovery and sleep post-exercise are core temperature reductions and parasympathetic re-activation (Douzi et al., 2018) however these mechanisms were unlikely to have had a significant influence in the current study.

### Study Limitations

As mentioned prior, the limitation of not assessing muscle damage markers beyond 24 h post-exercise should be noted. The peak muscle damage response post-downhill run is likely to occur between 24 and 48 h with several downhill run studies indicating closer to 24 h (Close et al., 2005; Crystal et al., 2013; Dolci et al., 2015; Rossato et al., 2015). It is thereby conceivable that the damage response at 24 h would be a reliable indicator of damage markers at 48 h and any alleviation at 24 h would likely result in quicker recovery to baseline (Haq et al., 2021).

Due to the logistics of transporting WBC participants for their treatments after the run and not being able to control all ambient conditions, it is possible that the Doppler ultrasound measures were not as reliable as anticipated. Since the cold water treatments occurred in the same building as the downhill run, the blood flow measures for these participants were likely to have been more reliable.

The reliability of the sleep measurements is also questionable, which could explain the lack of significant findings in this variable. Whilst previous studies have assessed sleep parameters post-WBC (Schaal et al., 2014; Douzi et al., 2018), this is the first cryotherapy study to use Fitbit watches. Wrist actigraphs have become a more popular mode in the assessment of sleep (Tabben et al., 2018). Polysomnography has also been utilized to good effect (Robey et al., 2013). Due to the multi-objective nature of the study, such invasive assessment of participants' sleep would have arguably been impractical and ecologically invalid.

Additionally, only one marker of inflammation was assessed due to constraints on timing and resources, with insignificant findings. Whilst the relevance of IL-6 for muscle damage and recovery has been established (Bruunsgaard et al., 1997; Dolci et al., 2015) other studies indicate that plasma IL-6 increases is not a reliable marker to assess EIMD severity (Hirose et al., 2004; Robson-Ansley et al., 2010). Measuring other inflammatory markers implicated in EIMD—e.g., IL-1β, IL-10, CRP, and TNF-α (Pournot et al., 2011; Krueger et al., 2018)—would provide more insight into the specific local and systemic inflammatory responses, their time course and potential interactions between different inflammatory molecules following muscle damaging exercise.

## Conclusions

Further insight is provided into the potential optimum WBC protocol for supporting post-exercise recovery and this is the first study to manipulate treatment timing as a factor. Delayed WBC treatments of several hours were ineffective in treating muscle damage, therefore indicating that it may be preferable to apply WBC within 1 h after exercise for optimum muscle strength recovery benefits. This may imply a positive outcome for busy athletes with demanding schedules, but could represent additional challenges to practitioners who do not have quick access to treatments. WBC treatment timing does not have a significant influence on post-exercise recovery parameters otherwise. Since WBC does not appear to be any more beneficial than CWI for post-downhill run recovery, CWI might be a preferred option due to its lower expense and relative ease of access. Additionally, there was no impact of either cold intervention on inflammation or sleep post-exercise. It would be beneficial for future research to focus more on comparing these two cold treatments following muscle damaging exercise bouts, particularly with regards to muscle swelling, blood flow and inflammatory markers, as well as investigate the impact of delayed treatments for CWI.

## Data Availability Statement

The raw data supporting the conclusions of this article will be made available by the authors, without undue reservation.

## Ethics Statement

The studies involving human participants were reviewed and approved by University of Northampton Graduate School Research Ethics Committee. The patients/participants provided their written informed consent to participate in this study.

## Author Contributions

AH, WR, and AB contributed to the study conception and design. AH performed the material preparation, data collection, data analysis, and wrote the first draft of the manuscript. Supervision by WR and AB. All authors reviewed and edited previous versions of the manuscript and read and approved the final version of the manuscript.

## Conflict of Interest

The authors declare that the research was conducted in the absence of any commercial or financial relationships that could be construed as a potential conflict of interest.

## Publisher's Note

All claims expressed in this article are solely those of the authors and do not necessarily represent those of their affiliated organizations, or those of the publisher, the editors and the reviewers. Any product that may be evaluated in this article, or claim that may be made by its manufacturer, is not guaranteed or endorsed by the publisher.
